# Associations of Serum CXCL12α and CK Levels with Skeletal Muscle Mass in Older Adults

**DOI:** 10.3390/jcm12113800

**Published:** 2023-05-31

**Authors:** Ze Chen, Thea Laurentius, Yvonne Fait, Aline Müller, Eva Mückter, Leo Cornelius Bollheimer, Mahtab Nourbakhsh

**Affiliations:** Department of Geriatric Medicine, RWTH Aachen University Hospital, 52074 Aachen, Germany; zchen@ukaachen.de (Z.C.); tlaurentius@ukaachen.de (T.L.); ifait@ukaachen.de (Y.F.); almueller@ukaachen.de (A.M.); emueckter@ukaachen.de (E.M.); cbollheimer@ukaachen.de (L.C.B.)

**Keywords:** skeletal muscle, inflammation, cytokines, chemokines, geriatrics, creatine kinase

## Abstract

Sarcopenia, a condition characterized by gradual loss of skeletal muscle mass and function, is a complex diagnosis; the decisive criterion in this diagnosis is the measurement of appendicular skeletal muscle index (ASMI). To identify potential serum markers predictive of sarcopenia in older adults, we evaluated correlations between ASMI, clinical data, and 34 serum inflammation markers in 80 older adults. Pearson’s correlation analyses confirmed that ASMI was positively correlated with nutritional status (*p* = 0.001) and serum creatine kinase (CK) (*p* = 0.019) but negatively correlated with serum CXCL12α (*p* = 0.023), a chemoattractant for muscle stem cells. In the case group, ASMI was negatively correlated with serum interleukin (IL)-7 (*p* = 0.024), a myokine expressed and secreted from skeletal muscle cells in vitro. Multivariate binary logistic regression analyses identified four risk factors for sarcopenia in our study: advanced age (*p* = 0.012), malnutrition (*p* = 0.038), low serum CK levels (*p* = 0.044), and high serum CXCL12α levels (*p* = 0.029). Low CK and high CXCL12α levels serve as combinatorial serum markers of sarcopenia in older adults. The linear correlation between ASMI and CXCL12α levels may facilitate the development of new regression models for future studies on sarcopenia.

## 1. Introduction

Sarcopenia is characterized by the progressive and generalized loss of skeletal muscle strength, mass, and function; these changes are accompanied by considerable risks of physical disability, poor quality of life, and death [1,2]. Muscle mass loss has long been considered a geriatric syndrome, and sarcopenia was not recognized as an independent disease with major social and economic burdens to individuals and society until 2016 [3]. The reported prevalence of sarcopenia varies among study populations; therefore, its diagnostic criteria and evaluation methods may require reasonable adjustments within individual studies [4]. It is thus difficult to define generalized cut-off points for loss of skeletal muscle mass, which is commonly estimated by scores on the appendicular skeletal muscle index (ASMI) based on dual-energy X-ray absorptiometry (DXA) or bioelectrical impedance analysis (BIA) [5]. DXA is widely used for standard reference measurements of lean muscle mass in young athletes. However, there are some disadvantages associated with DXA which limit the routine application of DXA for observational studies of older adults, including radiation exposure, non-portability, and the need for specialized personnel. Although BIA minimally overestimates lean muscle mass compared to DXA, recent studies have demonstrated that BIA measurements are highly accurate and reliable for muscle mass evaluation in older adults [6]. To encourage research and development, the European Working Group on Sarcopenia in Older People (EWGSOP) has provided ranges of ASMI cut-off points for the diagnosis of sarcopenia based on DXA and BIA [1].

Ageing may be the most relevant contributing factor to the loss of skeletal muscle mass and function. A previous study revealed that advanced age (>65 years) is significantly associated with sarcopenia [7]. After the age of 50 years, human skeletal muscle mass and strength decrease by 1–2% per year. This age-related weakening has been exclusively attributed to decreases in the number and length of fast-twitch muscle fibers, which provide quick and powerful forces [8]. A clinical indicator of skeletal muscle damage is the blood serum level of creatine kinase (CK) [9]. Creatine kinase (CK) is the enzyme responsible for catalyzing the exchange of phosphates in the creatine/phosphocreatine shuttle. In addition to mitochondrial CK genes, three genes express disparate CK isomers either ectopically or in brain and muscle cells. In human skeletal muscle, the CK pool is normally composed of 90% muscle CK (CK-MM) and 10% heart CK (CK-MB). By muscle damage (e.g., rhabdomyolysis), extreme exercise, and statin-induced myositis, serum CK can be raised by up to 10 times [10]. Serum CK levels of 55–170 U/L in males and 30–135 U/L in females are considered normal in persons of Northern European descent (>65 years). In addition to neuromuscular disorders, other conditions can also cause elevated creatine kinase, such as hyperparathyroidism, Cushing syndrome, hyponatremia, and medications [11]. Accordingly, serum CK levels are monitored in various clinical studies of seizure, coronary artery disease, colitis, hyperlipidemia, anesthesia, and rehabilitation training and exercise (https://clinicaltrials.gov) (accessed on 3 May 2023). Furthermore, elevated CK can also be an incidental finding in patients without muscle-related symptoms [11]. Serum CK levels have not yet been systematically analyzed in older people with sarcopenia.

The Mini Nutritional Assessment (MNA) is the preferred method of evaluating malnutrition in older adults; malnutrition is recognized as another factor that contributes to sarcopenia [12]. A high-quality diet was found to have a beneficial effect on skeletal muscle function [10,13]. Moreover, several studies reported the importance of sufficient intakes of protein, vitamin D, and antioxidants for the early prevention of sarcopenia and promotion of physical capability in older age [14,15]. Although much of this evidence is observational, the prevalence of low nutrient intake and poor nutritional status among older adults with sarcopenia has made malnutrition a current concern.

Chronic inflammation has also been implicated in the pathogenesis of sarcopenia in various studies [16]. Elevated levels of pro- and anti-inflammatory factors, such as interleukin (IL)-6, tumor necrosis factor (TNF)-α, and heat shock protein 27 (HSP27), are associated with sarcopenia and frailty [17,18]. Inflammatory cytokines have been suggested to activate specific signaling pathways that reduce the levels of growth hormones (GH and IGF-1), resulting in reduced protein anabolism, increased skeletal muscle atrophy, and, finally, sarcopenia [19,20]. Moreover, low hand grip strength was correlated with higher levels of the inflammation markers IL-6 and C-reactive protein (CRP) in older adults [21]. These observations indicate a direct link between age-related deterioration of skeletal muscle and the immune system that manifests in sarcopenia and immune senescence, respectively [22].

The homeostatic balance of regenerative and degenerative processes is essential for maintaining skeletal muscle mass [23]. The regeneration of skeletal muscle tissue is governed by the recruitment and differentiation of skeletal muscle stem cells activated by muscle tissue degeneration induced by injuries, inflammation, or reactive oxygen species (ROS). Experimental studies have found that muscle stem cells are recruited to their target location through a variety of chemokines, including CXCL12α, which was initially implicated in the activation and migration of inflammatory cells [24]. This small chemokine is a splice variant of the CXCL12 precursor protein, which is also involved in multiple regulatory processes in embryogenesis, angiogenesis, and inflammation [25,26]. In a mouse model, CXCL12 and its downstream STAT3 signaling affected the expression of paired box protein-7 (PAX7) and myoblast determination protein 1 (MyoD) in transplanted human mesenchymal stem cells after muscle injury [27,28]. Moreover, transient STAT3 inhibition promoted satellite cell expansion and enhanced tissue repair in aged or dystrophic muscles of mice [29]. It was therefore hypothesized that chronic elevation of CXCL12 levels may compromise the myogenic differentiation and skeletal muscle tissue regeneration after injury through STAT3 signaling in human satellite cells [24]. However, a direct link between CXCL12α and sarcopenia has not yet been reported.

To identify potential biomarkers for sarcopenia, we established a comprehensive set of anthropometric characteristics, clinical data, and serum levels of 34 cytokines and chemokines in 80 older persons of Northern European descent who underwent a battery of geriatric assessments, including the Timed Up and Go (TUG) test, De Morton Mobility Index (DEMMI), Mini-Mental State Examination (MMSE), Frailty Index (FI), Barthel Index (BI), hand grip strength, Falls Efficacy Scale (FES), Mini Nutritional Assessment (MNA), fall history over the last six months, Strength, Assistance with Walking, Rising from a Chair, Climbing Stairs, and Falls (SARC-F) questionnaire, and the AMSI. Anonymized study data have been made available (https://www.ukaachen.de/sarkopenie, accessed on 3 May 2023) to facilitate advances in the diagnosis and prognosis of sarcopenia in older adults.

## 2. Materials and Methods

### 2.1. Study Participants

This cross-sectional study included 80 consecutive inpatients of a geriatric clinic in Germany between February and December 2019. Individuals were primarily included if they met the following criteria: over 65 years of age, able to understand simple instructions, able to walk 10 m with or without a walking aid, and voluntarily consented to participate in the study. The exclusion criteria were as follows: severe visual impairment, severe statin-induced myopathy, cardiovascular disorders, severe heart failure, implantable cardioverter defibrillator or pacemaker, or other reasons that would prevent the individual from completing the study. All participants in the current study were of Northern European descent.

### 2.2. Blood Sample Collection and Processing

Fasting blood samples were collected by venipuncture of the median cubital vein using commercially available collection tubes (BD Vacutainer^®^; Becton, Dickinson and Co., Franklin Lakes, NJ, USA). Blood samples were left at room temperature for 20 min. Serum was then separated by centrifugation at 1000× *g* for 10 min at 4 °C. Serum aliquots were stored at −80 °C until analysis. All variable measurements were performed in the same laboratory (MVZ Labor, Bochum, Germany) and on the same day as sampling.

### 2.3. Measurement of Candidate Biomarkers

The Cytokine & Chemokine Convenience 34-Plex Human Panel 1A (EPXR340-12167-901) (Thermo Fisher Scientific, Waltham, MA, USA) was used to assess the levels of the 34 following human inflammatory markers in the serum samples: granulocyte-macrophage colony-stimulating factor (GM-CSF), interferon (IFN)-α, IFN-γ, IL-1α, IL-1β, IL-1RA, IL-2, IL-4, IL-5, IL-6, IL-7, IL-8 (CXCL8), IL-9, IL-10, IL-12p70, IL-13, IL-15, IL-17A, IL-18, IL-21, IL-22, IL-23, IL-27, IL-31, TNF-α, TNF-β, eotaxin, CXCL1α, CXCL10, CCL2, CCL3, CCL4, CCL5, and CXCL12α. Sample preparation, assays, and analyses were performed as described in the manufacturer’s instructions. The concentration of each marker was determined as the mean value of duplicate measurements. Reads under the assay detection levels or exceeding three times the SD of the mean (outliers) were excluded. 

### 2.4. Grip Strength Measurement

Hand grip strength was measured by a SAEHAN DHD-1 digital hand dynamometer (SAEHAN Corporation, Masan, Republic of Korea). The participant was seated upright on a chair with their feet firmly placed on the ground and their elbow entirely resting on the armrest at a 90° angle. The dynamometer was adjusted to fit the participant’s hand. After instruction, the participant pressed the dynamometer as hard as possible (i.e., displaying their maximum strength), and the results were recorded in kilograms to one decimal place. The test was performed 3 times with the dominant hand, and the average value was recorded.

### 2.5. Bioelectrical Impedance Analysis

A BIA device (BIACORPUS RX 4004M; MEDI CAL Healthcare GmbH, Karlsruhe, Germany) was used to estimate the appendicular skeletal muscle mass (ASMM). The integrated software of the BIA device was then used to calculate the ASMI based on the Sergi equation, as described previously [2]. Measurements were performed in a supine position in a standard hospital bed (maximum inclination of the head: 30°). The patient rested in a supine position for 5 min before measurements were initiated. Prior to the measurements, the patient’s hands and heels were moisturized using a disinfecting standard towel. Two electrodes were placed on each extremity. The proximal electrode was placed on the upper border of an imaginary line between the radius and ulna head and between the medial and lateral malleoli. Distal electrodes were placed within 5 cm of the distal border of the proximal electrodes. Whenever possible, measurement was performed bilaterally. The temperature (18–22 °C) and humidity (50–60%) of the measuring room were kept constant. The participants were instructed to be properly hydrated and not to eat 3–4 h before measurements.

### 2.6. Statistical Analysis

The data were analyzed using SPSS 21.0 software (IBM Corp., Armonk, NY, USA). Descriptive statistics were applied to characterize the study groups and to test the normal distribution of ASMI. Continuous variables were compared with the independent sample *t*-test or Wilcoxon rank-sum test, as appropriate, and expressed as the mean ± standard deviation (SD). Categorical variables were compared using the Pearson’s chi-square test or Fisher’s exact test and expressed as counts or percentages with 95% confidence intervals (CIs). Independent sample *t*-test was used to determine the significance (*p*) or the quantity (*t*-value) of differences between two groups that followed normal distribution. Pearson’s correlation analysis was conducted to identify associations between ASMI scores and the other variables of the study. For multivariate and univariate analyses, binary logistic regression analysis and Pearson’s chi-square tests were conducted to estimate the statistical probability (odds ratio (OR)) that the independent variables age, MNA scores, CK levels, and CXCL12α levels can predict the ASMI of the case group.

## 3. Results

### 3.1. Association of Skeletal Muscle Mass Decline with CK and CXCL12α

**Figure 1:**
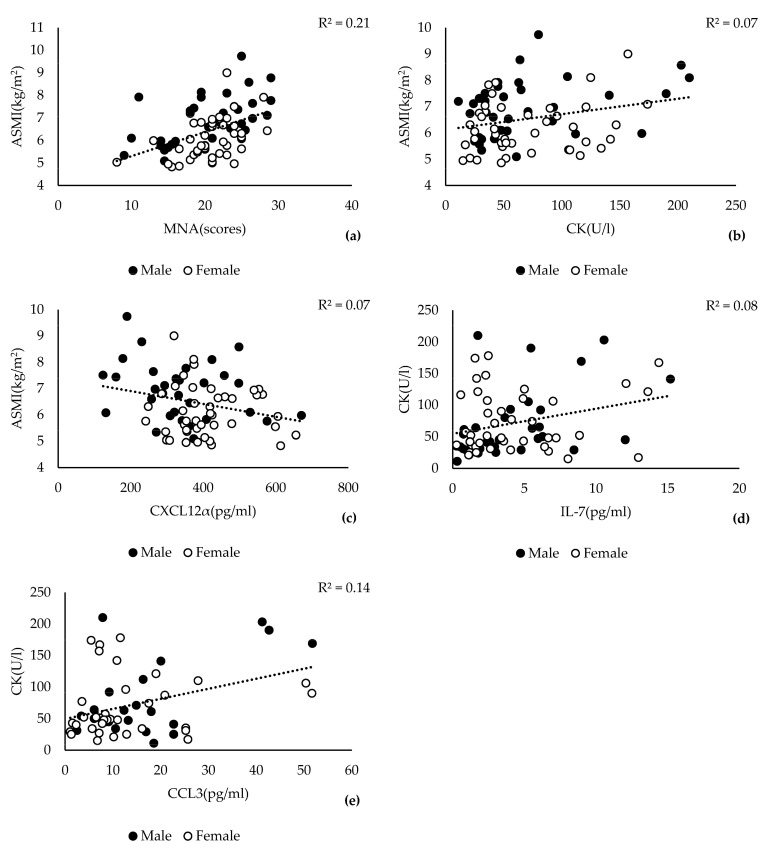
Relationships among ASMI scores (**a**–**c**), MNA scores (**a**), CK levels (**b**,**d**,**e**), and inflammatory markers CXCL12 (**c**), IL-7 (**d**), and CCL3 (**e**) within the entire study population (*n* = 80). Pearson’s correlation analysis was performed using entire study data available at https://www.ukaachen.de/sarkopenie, accessed on 3 May 2023. Pearson’s correlation coefficient (R^2^) and the significance (*p*) are provided on the top of each graph. The examined variables and their units are indicated on the corresponding axis. Male and female participants are indicated by black and white circles, respectively. The linear regression is represented by the dotted line in each graph.

The descriptive characteristics of the study participants expectedly revealed significant differences between the male and female participants in weight, hand grip strength, and body fat rate (BFR) and a moderate difference in the mean ASMI scores (Supplementary Table S1). We performed Pearson’s correlation analysis to examine the linear relationships between ASMI scores and 66 different variables, including 34 serum markers. Consistent with previous reports, we found a significant positive correlation between ASMI and MNA scores (Figure 1a, r = 0.416, *p* = 0.001). In addition, we discovered a positive correlation of serum CK levels (r = 0.264, *p* = 0.019) and a negative correlation of CXCL12α levels (r = −0.264, *p* = 0.023) with ASMI scores in our study (Figure 1b,c). All 63 other variables showed no correlation with ASMI scores in Pearson’s correlation analysis (Supplementary Table S2). Because elevated CK levels were previously linked to skeletal muscle inflammation [11], we examined the correlations between serum CK levels and inflammatory markers. Indeed, CK levels were positively correlated with IL-7 (Figure 1d, r = 0.286, *p* = 0.015) and CCL3 (Figure 1e, r = 0.371, *p* = 0.004) in our study. Pearson’s correlation analyses of male or female groups revealed weaker and statistically insignificant correlations in female or male groups with a smaller number of participants (Supplementary Table S3). Thus, it seems unlikely that sex had a significant impact on the reported correlations reported here.

### 3.2. Evaluation of ASMI Cut-Offs for the Sarcopenia Case Group

The ASMI scores in our study followed a normal distribution in the male and female participants (Supplementary Figure S1). For the assignment of study groups in accordance with EWGSOP2 guidelines, we considered ASMI cut-offs of ≥7 kg/m^2^ in males and ≥5.5 kg/m^2^ as normal levels (Supplementary Figure S1). Using independent *t*-test, the comparison of the study groups’ characteristics showed that only sex, weight, and MNA scores were significantly different (Table 1). All 63 other variables were not significantly different between the two groups (Supplementary Table S4). Previous studies reported that BIA tends to overestimate ASMI scores and suggested adjusting ASMI cut-offs based on the study data distribution accordingly [30,31]. Therefore, we applied independent *t*-test to evaluate ASMI cut-offs in our study based on the magnitude of differences (*t*-value) in a dependent variable (MNA score) and the number of divergent variables between the projected study groups. The highest difference in MNA scores was obtained at ASMI cut-offs of 7.3 kg/m^2^ in male and 6.8 kg/m^2^ in female participants (Supplementary Figure S1). Consistently, the number of significantly divergent variables excelled at ASMI cut-offs of 7.3 kg/m^2^ in male and 6.8 kg/m^2^ in female participants (Supplementary Figure S1). Independent *t*-test revealed significant differences in six variables between the case (males < 7.3 kg/m^2^ and females < 6.8 kg/m^2^) and control (males ≥ 7.3 kg/m^2^ and females ≥ 6.8 kg/m^2^) groups (Table 1). Together, the case group was most significantly associated with lower weight, MNA scores, serum IL-7, and CK and with higher BFR and monocyte counts in our study. The effect sizes of differences varied among the variables ranging from small (age, r = 0.224) to medium (weight, r = 0.485) (Supplementary Table S5).

### 3.3. Low ASMI Is Significantly Associated with Lower CK and IL-7 Levels

**Figure 2:**
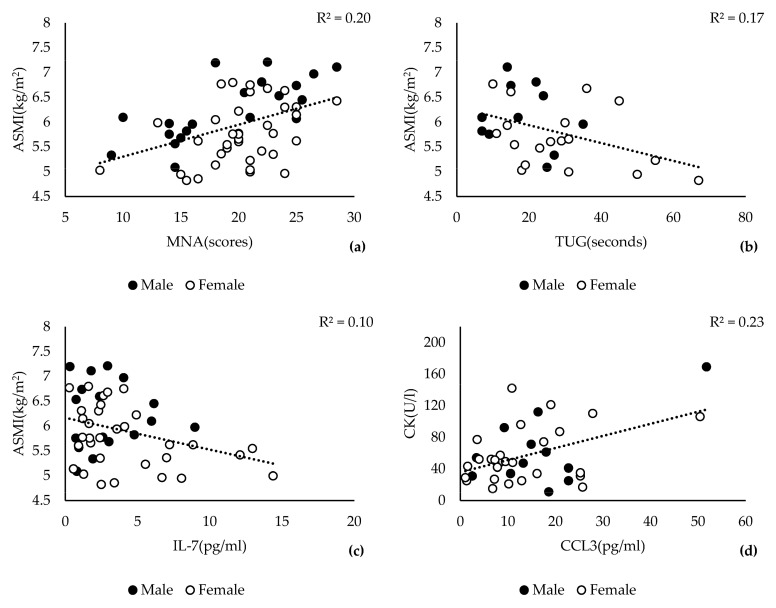
Relationships among ASMI scores (**a**–**c**), MNA scores (**a**), CK levels, TUG (**c**), and inflammatory markers IL-7 (**b**) and CCL3 (**d**) within the sarcopenia case group (males: ASMI score < 7.3 kg/m^2^ and females: AMSI score < 6.8 kg/m^2^, *n* = 56). Pearson’s correlation analysis was performed using entire study data available at https://www.ukaachen.de/sarkopenie accessed on 3 May 2023. Pearson’s correlation coefficient (R^2^) and the significance (*p*) are provided on the top of each graph. The examined variables and their units are indicated on the corresponding axis. Male and female participants are indicated by black and white circles, respectively. The linear regression is represented by the dotted line in each graph.

For further examination of the case group characteristics, we explored possible linear relationships between different variables using Pearson’s correlation analysis. In the case group, ASMI scores were significantly correlated with MNA scores (r = 0.449, *p* = 0.001), followed by TUG scores (r = −0.408, *p* = 0.028) and IL-7 levels (r = −0.322, *p* = 0.024) (Figure 2a–c). The CK level was positively correlated with the CCL3 level in the case group (r = 0.345, *p* = 0.023) and in the entire study population described above (Figure 2d).

### 3.4. Serum Levels of CK and CXCL12α Are Markers for Sarcopenia

The association of ASMI with serum levels of CXCL12α and CK prompted us to explore their use as risk factors for the case group. Preliminary Pearson’s chi-square tests indicated a potential influence of CXCL12α levels > 240 pg/mL for the case group in our study (Supplementary Table S6). However, binary multivariate logistic regression analysis is frequently used to predict the relationships between dependent and independent variables and estimate the probability for a condition depending on multiple sets of variables. We used this approach based on the normative ASMI cut-offs (ASMI scores < 7.3 kg/m^2^ in males and <6.8 kg/m^2^ in females). The results identified age > 83 years (OR = 27.571, 95% CI: 2.044–371.845, *p* = 0.038), MNA scores ≤ 24 (OR = 5.776, 95% CI: 1.106–30.161, *p* = 0.012), CK levels ≤ 120 U/L (OR = 6.439, 95% CI: 1.053–39.362, *p* = 0.044), and CXCL12α levels > 240 pg/mL (OR = 19.131, 95% CI: 1.349–271.377, *p* = 0.029) as influencing risk factors for the case group in our study (Table 2). Thus, these results suggest that CK and CXCL12α levels may serve as additional markers in the assessment or diagnosis of sarcopenia in older adults.

## 4. Discussion

One of the main challenges in translational medicine is identifying reliable biomarkers to differentiate case and control groups or to develop early diagnostics. This task becomes even more difficult in age-related and multifactorial conditions, such as sarcopenia. In the present study, we identified serum levels of CK and CXCL12α as potential serum markers of sarcopenia in older adults. Despite our efforts to use the ASMI cut-off values recommended by the EWGSOP2, careful adjustments were necessary to identify sarcopenia cases and to facilitate the findings of our study. Previous reports suggested that the application of static ASMI cut-off values may significantly affect the prevalence of sarcopenia and thus conclusions from observational studies [30,32,33]. Therefore, placing a higher priority on adjusting the specific ASMI cut-off values based on normative data can significantly improve their diagnostic value in future studies on sarcopenia. 

Another motivation for adjusting the ASMI cut-off values in our study originated from the limitations of BIA-based estimations of skeletal muscle mass. Although recommended by the EWGSOP, BIA-based estimations are sensitive to the water and electrolyte contents of the body. Moreover, older participants are particularly prone to underhydration. Further limitations may result from BIA algorithms, which are often developed by manufacturers based on measurements of healthy and young volunteers and are thus not descriptive for older adults [5]. Advances in radiological imaging modalities, such as dual-energy X-ray absorptiometry (DXA), computed tomography (CT), magnetic resonance imaging (MRI), and ultrasounds (USs), can be used to evaluate muscle mass and quality. However, all these methods impose specific limitations and require certain adjustments for assessing muscle mass in older adults [5].

**Figure 3:**
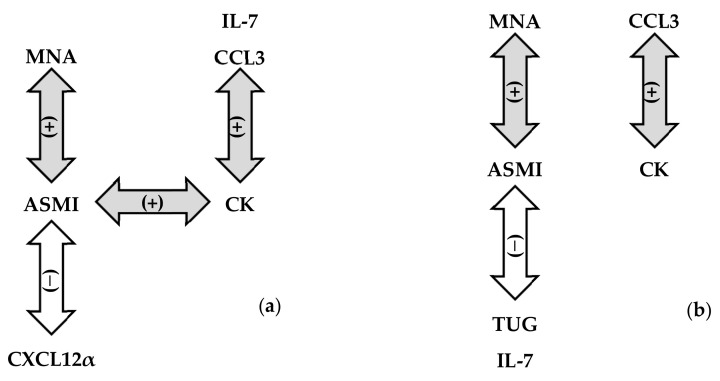
Schematic summary of the correlations between ASMI scores and other variables in all participants (**a**) and those in the sarcopenia case group (males: ASMI score < 7.3 kg/m^2^ and females: AMSI score < 6.8 kg/m^2^ (**b**). Positive and negative correlations are depicted by gray and white bidirectional arrows, respectively, with (−) or (+).

Despite the limited number of participants in our study, the case group had a consistent correlation between ASMI scores and two well-recognized diagnostic factors for sarcopenia, MNA and TUG scores [1]. Furthermore, MNA scores of 24 or less and an age higher than 83 years were identified as influencing factors of the case group. Thus, the use of normative ASMI cut-off values for male and female participants was justified and allowed the identification of three additional serum markers for muscle mass decline in older adults: low CK levels, high IL-7 levels, and high CXCL12α levels. Furthermore, serum levels of CCL3 and CK were positively correlated in older adults. Thus, the CCL3 level may serve as a supporting marker to confirm cases of low CK levels associated with muscle mass decline. To provide a better overview of the findings, we schematically summarized all detected correlations within the entire population as well as those within the designated sarcopenia case group (Figure 3a and Figure 3b, respectively).

CXCL12α was the most relevant serum marker for sarcopenia in our study. First, CXCL12α serum levels were inversely correlated with the ASMI scores of 80 older adults (aged 69–93 years). Second, binary logistic regression analysis of sarcopenia cases identified CXCL12α levels > 240 pg/mL as one of the risk factors for sarcopenia in our study. Previous animal studies have implicated CXCL12α in the pathogenesis of osteoporosis and sarcopenia [34,35]. However, possible correlations of sarcopenia with serum CXCL12α levels have not yet been monitored or reported in human clinical studies. Interestingly, elevated plasma CXCL12α was previously associated with lower bone density and increasing age, which is often accompanied by sarcopenia [36,37]. In our study, however, serum CXCL12α did not correlate with increasing age (r = −0.103, *p* = 0.374) or any other variables. Nevertheless, skeletal muscles and bones are closely connected at multiple levels (anatomically, functionally, mechanically, and metabolically). Therefore, a causal link between increased serum CXCL12α levels and sarcopenia may consequently lead to lower bone density with ageing. In experimental studies, elevated CXCL12α was found to activate STAT3 signaling through binding to its receptor CXCR4 in skeletal muscle stem cells and inhibit the expression of PAX7 and MyoD [24,26,34]. Thus, a possible mechanism of anti-myogenic action was hypothesized for CXCL12 in muscle stem cells and sarcopenia [24]. Although our results are in accordance with this hypothesis, elevated serum CXCL12α may not necessarily reflect its level or effect in ageing muscle tissue, and future investigations are required to explore the relationship between CXCL12α levels in serum and in skeletal muscle tissue of older adults.

Over the years, the serum CK level has been used as an indicator of muscle activity. However, previous observational studies on sarcopenia did not observe or report a correlation between serum CK levels and ASMI scores. This discrepancy may be due to numerous factors that can affect serum CK levels, including race, age, medications, physical activity, and injuries [11]. A recent study on Asian patients with osteoarthritis associated increased serum CK levels with elevated skeletal muscle activity [38]. This finding is consistent with the positive correlation between serum CK levels and ASMI scores in the present study (Figure 1b) as well as the results from our logistic regression analysis, which identified CK levels < 120 U/L as a risk factor for sarcopenia. Furthermore, CK levels were positively associated with serum levels of IL-7 and CCL3 (Figure 1c,d), which have been previously implicated in the regulation of human skeletal muscle stem cells and muscle tissue regeneration [39,40]. Thus, increased skeletal muscle mass, activity, and subsequent microscopic injuries may lead to elevated serum levels of CK. Despite its significant association with skeletal muscle mass, CK levels will likely play a restricted role in the future diagnosis of sarcopenia, particularly in older adults, who often have multiple conditions or medications that may adversely alter CK levels. These confounding factors may also explain why we found no correlation between ASMI scores and CK levels in the sarcopenia case group.

In conclusion, our study found that skeletal muscle mass decline is associated with elevated CXCL12α levels and decreased CK levels in the serum of older adults. Multiple conditions can affect serum CK levels and may restrict its diagnostic value for low muscle mass in older adults. However, CXCL12 may serve as a diagnostic biomarker for skeletal muscle mass decline in older adults.

## Figures and Tables

**Table 1:** Characteristics of study groups based on different ASMI cut-off values analyzed using descriptive statistics.

	Normal ASMI Male ≥ 7.0 kg/m^2^ Female ≥ 5.5 kg/m^2^ (*n* = 49)	Low ASMI Male < 7.0 kg/m^2^ Female < 5.5 kg/m^2^ (*n* = 31)	*p*	Control Group Male ≥ 7.3 kg/m^2^ Female ≥ 6.8 kg/m^2^ (*n* = 24)	Case Group Male < 7.3 kg/m^2^ Female < 6.8 kg/m^2^ (*n* = 56)	*p*
Age (years)	80.73 ± 6.27	82.58 ± 6.57	>0.05	79.46 ± 5.81	82.30 ± 6.52	>0.05
Sex (F/M)	32/17	13/18	0.041	10/14	35/21	>0.05
ASMI (kg/m^2^)	7.01 ± 0.99	5.67 ± 0.63	<0.001	7.79 ± 0.70	5.93 ± 0.65	<0.001
Weight (kg)	76.78 ± 15.47	62.22 ± 10.65	<0.001	81.81 ± 13.39	66.56 ± 14.07	<0.001
BFR (%)	34.41 ± 8.59	31.90 ± 7.15	>0.05	29.95 ± 8.37	34.93 ± 7.58	0.011
MNA (scores)	25.28 ± 16.07	18.52 ± 4.92	0.026	22.83 ± 4.27	19.80 ± 4.57	0.012
Monocyte (%)	9.06 ± 4.21	8.53 ± 2.86	>0.05	7.73 ± 2.39	9.34 ± 4.09	0.042
IL-7 (pg/mL)	4.35 ± 3.71	4.33 ± 3.67	>0.05	5.63 ± 4.06	3.74 ± 3.34	0.040
CK(U/L)	73.94 ± 52.44	69.13 ± 46.60	>0.05	91.38 ± 59.27	63.80 ± 43.50	0.017

**Table 2:** Influencing factors on sarcopenia case group detected using multivariate binary logistic regression analyses.

	OR	95% CI	*p*
Age (years)			
≤83	1.00		
>83	27.57	2.04–371.85	0.012
MNA (scores)			
>24	1.00		
≤24	5.78	1.11–30.16	0.038
CK (U/L)			
>120	1.00		
≤120	6.44	1.05–39.36	0.044
CXCL12α (pg/mL)			
≤240	1.00		
>240	19.13	1.35–271.38	0.029

## Data Availability

The data presented in this study are openly available at https://www.ukaachen.de/sarkopenie (accessed on 3 May 2023).

## Supplementary Materials

The following supporting information can be downloaded at: https://www.mdpi.com/article/10.3390/jcm12113800/s1, Figure S1: Distribution of ASMI scores among male and female study participants; Table S1: General characteristics of the study participants calculated using descriptive statistics; Table S2: Correlations between ASMI scores and all variables calculated using Pearson’s correlation analyses; Table S3: Correlations in male, female and all participants groups calculated using Pearson’s correlation analysis; Table S4: Characteristics of study groups based on EWGSOP ASMI cutoff values calculated using descriptive statistics; Table S5: Characteristics of study groups based on different ASMI cut-off values analyzed using descriptive statistics, Cohen’s d ($cohen^{\prime }s\;d=\frac{\left({\mathrm{Mean}}_{1}-{\mathrm{Mean}}_{2}\right)}{\sqrt{\left[\left(\sigma_{1}^{2}+\sigma_{2}^{2}\right)/2\right]}}$), and effect-size ($effect\;size\;r=\frac{\;d}{\sqrt{\left(d^{2}+4\right)}}$); Table S6: Influencing factors on sarcopenia case group analyzed using Pearson’s chi square tests.
