# Supplementary material for: Associations of Serum CXCL12α and CK Levels with Skeletal Muscle Mass in Older Adults
Source: J Clin Med. 2023 May 31;12(11):3800. doi: 10.3390/jcm12113800 (PMC10253690; doi:10.3390/jcm12113800)
Supplement: Supplementary file 1 [file jcm-12-03800-s001.zip › jcm-2407467-supplementary.pdf]

Figure S1 Distribution of ASMI scores among male and female study participants.

Using descriptive statistics, ASMI scores were plotted against the number of male (**a**) and female (**b**) participants. The mean values (Mean), standard deviation (Std. Dev.), and the total number of male (left) and female (right) participants are provided at the top of each graph. Dotted lines indicate the ASMI cut-offs according to the EWGSOP2 guidelines (7.0 kg/m^2 and 5.5 kg/m^2 in male and female participants, respectively). Solid lines indicate the normative ASMI cut-offs, which were used to assign case and control groups in the present study (7.3 kg/m^2 and 6.8 kg/m^2 in male and female participants, respectively). The 3D diagram shows the magnitude of differences in MNA scores expressed as t values (colour scale) based on different ASMI cut-offs in males (y-axis, blue) and females (**c**). The 3D diagram shows the number of significantly divergent variables ($p < 0.05$, magenta dots) in males (y-axis, blue) and females (x-axis, red) based on different ASMI cut-offs (**d**).

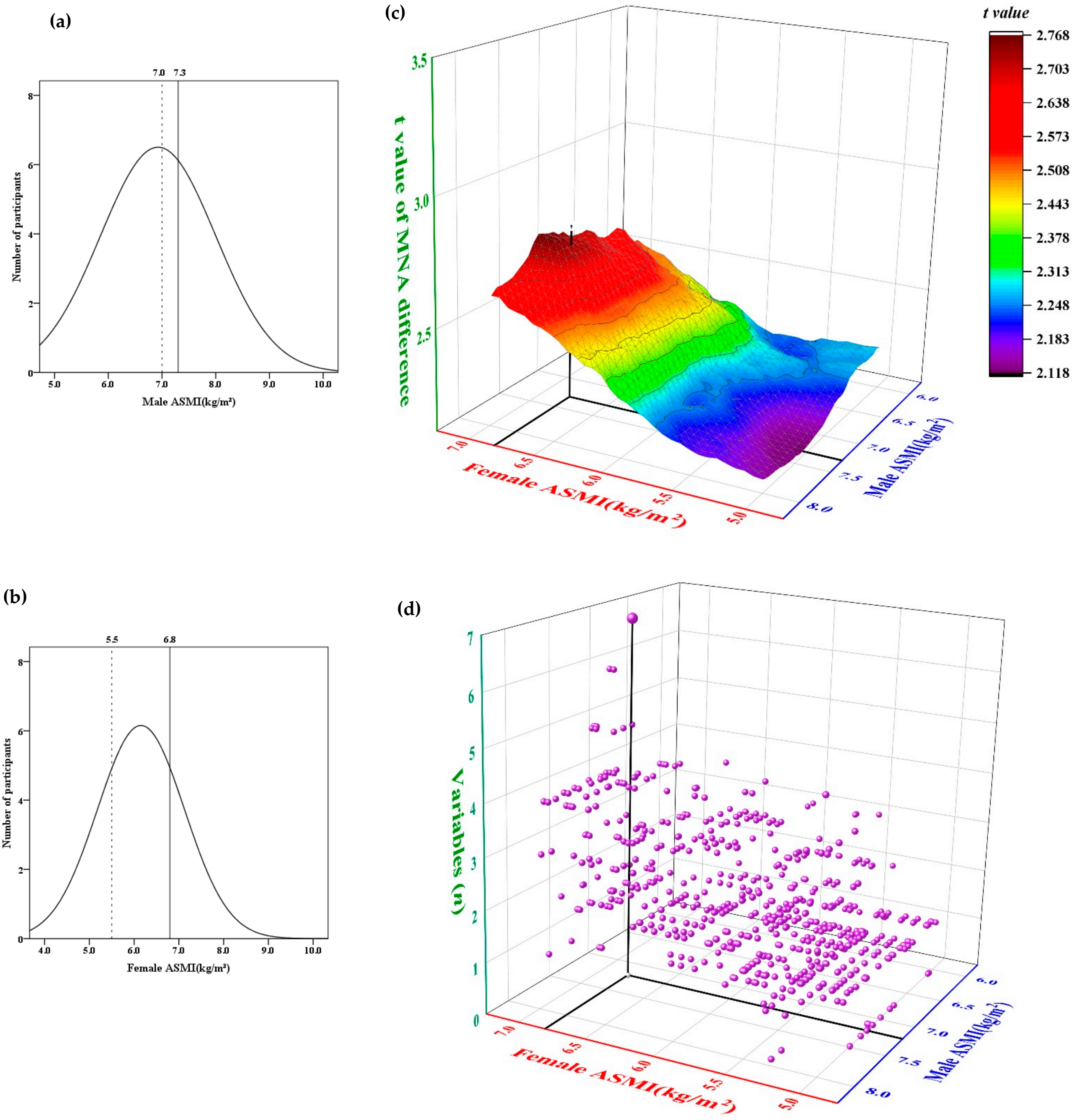

Table S1. General characteristics of the study participants calculated using descriptive statistics

	**Male (*n* = 35)**	**Female (*n* = 45)**	*p*
Age(years)	80.34±7.05	82.31±5.81	0.175
ASMI(kg/m²)	6.93±1.07	6.15±0.97	0.001
Weight(kg)	76.27±16.45	67.14±13.55	0.008
Hand Strength(kg)	20.64±10.04	13.84±5.61	0.001
BFR(%)	30.24±8.00	35.93±7.36	0.001
MNA(scores)	20.41±5.59	24.27±16.80	0.203
Sarc-F(scores)	5.11±1.41	4.56±1.58	0.104
CK(U/L)	69.77±53.24	69.28±43.77	0.965
Height(cm)	170.34±9.95	164.11±8.02	0.004
BMI(kg/m²)	26.21±4.92	25.38±4.16	0.416
TUG(s)	19.56±8.43	28.14±15.44	0.032
Demmi(scores)	38.58±21.76	37.53±13.81	0.811
MMSE(scores)	25.85±3.30	25.51±3.29	0.460
Frailty(scores)	2.74±1.07	2.98±0.94	0.299
Barthel(scores)	57.57±28.84	60.12±25.48	0.680
FES(scores)	32.66±9.79	36.11±9.99	0.126
Leukocytes(10^9/L)	7.27±2.40	7.99±2.84	0.233
Erythrocytes(10^{12}/L)	4.57±4.94	3.67±0.66	0.231
Hkt	37.27±13.30	33.21±6.62	0.081
Hemoglobin(g/dl)	11.77±3.52	11.37±3.02	0.580
MCV(fl)	90.48±11.49	90.28±13.52	0.946
MCH(pg)	32.98±1.35	31.41±9.41	0.529
MCHC(%)	32.98±1.35	32.50±1.21	0.109
Platelets(10^9/L)	245.56±98.32	299.44±92.79	0.015
Neutrophils(%)	67.92±10.58	67.96±8.43	0.988
Eosinophils(%)	3.18±2.72	3.16±2.80	0.978
Basophils(%)	0.61±0.33	0.68±0.36	0.468
Monocytes(%)	8.89±2.14	8.82±4.81	0.947

Lymphocytes(%)	19.65±9.11	18.90±6.73	0.731
Creatinine(mg/dl)	1.14±0.61	0.97±0.50	0.176
Ca(mmol/L)	2.18±0.13	2.19±0.17	0.888
IFN-α(pg/ml)	4.91±1.64	5.28±2.18	0.417
IFN-γ(pg/ml)	89.48±79.65	79.04±70.93	0.538
IL-1α(pg/ml)	4.52±11.91	6.01±27.62	0.775
IL-1RA(pg/ml)	3185.86±6183.20	2407.63±4025.79	0.578
IL-2(pg/ml)	6.81±12.37	4.70±5.16	0.385
IL-6(pg/ml)	41.32±54.30	22.52±28.31	0.289
IL-7(pg/ml)	4.35±3.66	4.33±3.73	0.981
IL-12p70(pg/ml)	2.48±1.48	2.74±2.49	0.620
IL-18(pg/ml)	78.89±58.62	76.59±58.59	0.864
CXCL1(pg/ml)	11.11±9.14	10.27±9.79	0.768
CCL24(pg/ml)	47.67±32.08	53.27±37.85	0.485
CXCL10(pg/ml)	28.90±19.34	37.89±36.34	0.192
CCL2(pg/ml)	57.44±43.17	56.92±51.84	0.966
CCL3(pg/ml)	16.03±12.91	13.10±11.94	0.375
CCL4(pg/ml)	136.45±218.73	142.04±300.56	0.927
CXCL12α(pg/ml)	341.36±124.62	412.09±100.55	0.008
CCL5(pg/ml)	20.17±5.94	20.16±6.12	0.994

Table S2. Correlations between ASMI scores and all variables calculated using Pearson's correlation analyses

	Correlation coefficient r	Level of significance *p*
Sex	0.367	0.001
Age	-0.241	0.031
Weight	0.652	<0.001
Height	0.123	0.279
BMI	0.714	<0.001
TUG	-0.228	0.158
Demmi	0.051	0.663
MMSE	-0.076	0.512
Frailty	-0.009	0.936
Barthel	0.138	0.227
Handgrip	0.248	0.029
FES	-0.136	0.230
MNA	0.416	0.001
SarcF	0.125	0.271
BFR	0.131	0.335
Leukocytes	0.079	0.487
Erythrocytes	0.027	0.815
Hkt	0.032	0.782
Hemoglobin	0.101	0.374
MCV	-0.013	0.912
MCH	0.046	0.685
MCHC	-0.083	0.475
Platelets	-0.217	0.055
Neutrophils	0.039	0.785

Eosinophils	0.053	0.708
Basophils	-0.051	0.717
Monocytes	-0.046	0.741
Lymphocytes	0.280	0.844
CK	0.264	0.019
Creatinine	0.027	0.817
Ca	0.142	0.282
IFN-α	0.050	0.660
IFN-γ	0.000	1.000
IL-1α	0.039	0.735
IL-1RA	0.161	0.245
IL-2	0.240	0.078
IL-6	0.231	0.247
IL-7	0.027	0.819
IL-12p70	-0.032	0.793
IL-18	0.059	0.606
CXCL1	-0.031	0.839
CCL24	-0.158	0.161
CXCL10	-0.157	0.163
CCL2	-0.150	0.220
CCL3	0.082	0.539
CCL4	-0.032	0.783
CXCL12α	-0.261	0.023
CCL5	-0.038	0.740

Table S3. Correlations in male, female and all participants groups calculated using Pearson's correlation analysis

Variables	Male (*n* = 35)		Female (*n* = 45)		All participants (*n* = 80)	
	R^2	*p*	R^2	*p*	R^2	*p*
ASMI, MNA	0.31	0.001	0.22	0.002	0.21	0.001
ASMI, CK	0.11	0.015	0.05	0.372	0.07	0.019
ASMI, CXCL12α	0.07	0.136	0.00	0.668	0.07	0.023
CK, IL-7	0.21	0.024	0.02	0.260	0.08	0.015
CK, CCL3	0.37	0.007	0.02	0.345	0.14	0.004

Table S4. Characteristics of study groups based on EWGSOP ASMI cutoff values calculated using descriptive statistics

	**normal ASMI**<br>Male ≥ 7.0 kg/m²<br>Female ≥ 5.5 kg/m²<br>(n = 49)	**low ASMI**<br>Male < 7.0 kg/m²<br>Female < 5.5 kg/m²<br>(n = 31)	p
Sex(F/M)	32/17	13/18	0.041
Age(years)	80.73±6.27	82.58±6.57	0.212
Weight(kg)	76.78±15.47	62.22±10.65	<0.001
Height(cm)	166.78±9.27	166.94±9.73	0.941
BMI(kg/m²)	27.96±3.95	22.23±2.72	<0.001
TUG(s)	22.61±10.09	26.53±16.88	0.365
Demmi(scores)	37.53±14.90	38.72±21.50	0.776
MMSE(scores)	2.32±0.63	2.52±0.57	0.152
Frailty(scores)	2.84±0.94	2.94±1.09	0.669
Barthel(scores)	60.21±27.23	57.10±26.70	0.620
Handgrip(kg)	17.12±8.34	16.11±8.70	0.608
FES(scores)	33.10±9.39	36.97±10.60	0.092
MNA(scores)	25.28±16.07	18.52±4.92	0.026
SarcF(scores)	4.80±1.47	4.81±1.62	0.976
ASMI(kg/m²)	7.01±0.99	5.67±0.63	<0.001
BFR(%)	34.41±8.59	31.907.15	0.180
Leukocytes(10^9/L)	8.03±2.51	7.11±2.84	0.131
Erythrocytes(10^{12}/L)	3.66±0.63	4.70±5.25	0.175
Hkt	33.29±6.44	37.67±14.03	0.064
Hemoglobin(g/dl)	11.36±2.91	11.84±3.71	0.527
MCV(fl)	90.90±13.18	89.54±11.84	0.643
MCH(pg)	31.50±9.09	33.38±18.08	0.541
MCHC(%)	32.51±1.12	33.00±1.47	0.104
Platelets(10^9/L)	278.24±95.70	273.00±104.05	0.820
Neutrophils(%)	67.41±9.60	68.73±9.35	0.626

Eosinophils(%)	3.20±2.58	3.11±3.02	0.912
Basophils(%)	0.63±0.35	0.67±0.35	0.684
Monocytes(%)	9.06±4.21	8.53±2.86	0.616
Lymphocytes(%)	19.73±7.79	18.56±8.25	0.274
CK(U/L)	73.94±52.44	62.00±38.84	0.291
Creatinine(mg/dl)	1.05±0.55	1.04±0.58	0.968
Ca(mmol/L)	2.18±0.17	2.20±0.12	0.600
IFN-α(pg/ml)	5.35±1.99	4.77±1.91	0.207
IFN-γ(pg/ml)	92.22±85.73	70.00±50.69	0.196
IL-1α(pg/ml)	7.36±28.67	2.46±4.32	0.349
IL-1RA(pg/ml)	3434.66±6101.96	1457±1085.99	0.169
IL-2(pg/ml)	6.78±10.59	3.38±2.96	0.169
IL-6(pg/ml)	34.81±51.66	29.83±32.63	0.787
IL-7(pg/ml)	4.35±3.71	4.33±3.67	0.979
IL-12p70(pg/ml)	2.68±2.39	2.56±1.60	0.823
IL-18(pg/ml)	84.42±64.83	66.68±44.63	0.192
CXCL1(pg/ml)	10.72±9.24	10.48±10.00	0.934
CCL24(pg/ml)	46.34±28.93	57.89±43.17	0.156
CXCL10(pg/ml)	37.24±34.78	28.75±21.44	0.226
CCL2(pg/ml)	56.00±48.34	59.09±49.18	0.800
CCL3(pg/ml)	14.12±11.73	14.60±13.62	0.888
CCL4(pg/ml)	140.86±297.63	137.62±211.88	0.959
CXCL12α(pg/ml)	382.14±114.47	380.21±121.10	0.944
CCL5(pg/ml)	20.67±5.97	19.36±6.07	0.345

Table S5. Characteristics of study groups based on different ASMI cut-off values analyzed using descriptive statistics, Cohen's d ($cohen's\ d = \frac{(\mathrm{Mean}_1 - \mathrm{Mean}_2)}{\sqrt{[(\sigma_1^2 + \sigma_2^2)/2]}}$), and effect-size ($effect\ size\ r = \frac{d}{\sqrt{(d^2+4)}}$).

	**normal ASMI Male≥7.0kg/m² Female≥5.5kg/m² (n=49)**	**low ASMI Male<7.0kg/m² Female<5.5kg/m² (n=31)**	*p*	**Cohen's d**	**effect-size(r)**	**control group Male≥7.3kg/m² Female≥6.8kg/m² (n=24)**	**case group Male<7.3kg/m² Female<6.8kg/m² (n=56)**	*p*	**Cohen's d**	**effect-size(r)**
Age(years)	80.73±6.27	82.58±6.57	>0.05	0.3	0.143	79.46±5.81	82.30±6.52	>0.05	0.5	0.224
ASMI(kg/m²)	7.01±0.99	5.67±0.63	<0.001	1.6	0.628	7.79±0.70	5.93±0.65	<0.001	2.8	0.809
Weight(kg)	76.78±15.47	62.22±10.65	<0.001	1.1	0.481	81.81±13.39	66.56±14.07	<0.001	1.1	0.485
BFR(%)	34.41±8.59	31.90±7.15	>0.05	0.3	0.157	29.95±8.37	34.93±7.58	0.011	0.6	0.298
MNA(scores)	25.28±16.07	18.52±4.92	0.026	0.6	0.274	22.83±4.27	19.80±4.57	0.012	0.7	0.324
Monocyte(%)	9.06±4.21	8.53±2.86	>0.05	0.1	0.073	7.73±2.39	9.34±4.09	0.042	0.5	0.234
IL-7(pg/ml)	4.35±3.71	4.33±3.67	>0.05	0.0	0.003	5.63±4.06	3.74±3.34	0.040	0.5	0.246
CK(U/l)	73.94±52.44	69.13±46.60	>0.05	0.1	0.048	91.38±59.27	63.80±43.50	0.017	0.5	0.256

Table S6. Influencing factors on sarcopenia case group analyzed using Pearson's chi square tests

		OR (95%CI)	*p*
Age (years)	>83<br>≤83	2.459(0.801, 7.548)	0.116
MNA (scores)	≤24<br>>24	3.308(1.168, 9.366)	0.024
CK (U/l)	>120<br>≤120	3.500(1.096, 11.147)	0.034
CXCL12α(pg/ml)	≤240<br>>240	15.588(1.701, 142.874)	0.015
